# Effects of desflurane and sevoflurane on somatosensory-evoked and motor-evoked potential monitoring during neurosurgery: a randomized controlled trial

**DOI:** 10.1186/s12871-021-01463-x

**Published:** 2021-10-07

**Authors:** Bingbing Xiang, Shulan Jiao, Yulong Zhang, Lu Wang, Yuting Yao, Feng Yuan, Rui Chen, Qijun Zhou

**Affiliations:** 1 Department of Anesthesiology, Chengdu Fifth People’s Hospital, Chengdu, 611130 China; 2 Department of Anesthesiology, The Second Affiliated Hospital of Kunming Medical University, Kunming, 650101 People’s Republic of China; 3 Department of Anesthesiology, Kunming Children’s Hospital, Kunming, 650101 China; 4 Department of Anesthesiology, First People’s Hospital of Yunnan Province, Kunming, 650101 China; 5 Department of Anesthesiology, Second Affiliated Hospital of Chongqing Medical University, Chongqing, 401331 China

**Keywords:** Monitoring, Somatosensory-evoked potentials, Motor-evoked potentials, Desflurane Sevoflurane

## Abstract

**Background:**

Better protection can be provided during neurosurgery due to the establishment of somatosensory-evoked potential (SEP) and motor-evoked potential (MEP) monitoring technologies. However, some studies have showed that inhaled halogenated anesthetics have a significant impact on neurophysiological monitoring.

**Methods:**

A total of 40 consecutive patients undergoing neurosurgery were randomly assigned to two groups receiving inhaled anesthetics, either desflurane or sevoflurane. Multiples levels (concentrations of 0.3, 0.6 and 0.9) of anesthetics were administered at minimum alveolar concentration (MAC), and then the latencies and amplitudes of SEPs and MEPs were recorded.

**Results:**

SEP and MEP signals were well preserved in patients who underwent neurosurgery under general anesthesia supplemented with desflurane or sevoflurane at concentrations of 0.3, 0.6 and 0.9 MAC. In each desflurane or sevoflurane group, the amplitudes of SEPs and MEPs decreased and the latencies of SEPs were prolonged significantly as the MAC increased (*P* < 0.05). The SEP latencies of both the upper and lower limbs in the desflurane group were significantly longer, and the SEP amplitudes were significantly lower than those in the sevoflurane group (*P* < 0.05). The MEP amplitudes in the desflurane group were significantly lower than those in the sevoflurane group (*P* < 0.05), only the amplitudes of the upper limbs at 0.3 MAC did not vary significantly.

**Conclusions:**

SEPs and MEPs were inhibited in a dose-dependent manner by both desflurane and sevoflurane. At the same MAC concentration, desflurane appeared to have a stronger inhibitory effect than sevoflurane. All patients studied had normal neurological examination findings, hence, these results may not be applicable to patients with preexisting deficits.

**Trial registration:**

The study registered on the Chinese Clinical Trial Registry (www.chictr.org.cn), Clinical Trials identifier ChiCTR2100045504 (18/04/2021).

## Background

Neurophysiological monitoring has a history of over 30 years and has been widely used in surgery for spinal cord, skull base, functional area tumors and epilepsy. Clinical methods for monitoring the nervous system include the wake-up test, somatosensory-evoked potentials (SEPs) and motor-evoked potentials (MEPs). The wake-up test is recommended as the golden standard, and can directly reflect the function of the nervous system during surgery. However, this test usually takes considerable time during surgery and frequently leads to psychological disorders in patients. Additionally, it cannot be used for continuous intraoperative monitoring and cannot detect suspicious nerve damage in time. Wake-up tests cannot be performed in patients with neuromuscular disease, weak muscle strength, and uncooperative behavior, nor in children and patients with mental disorders. SEP monitoring is currently a very popular method in neurosurgery, that can reflect the full function of the somatosensory conduction pathway. Decreased amplitudes or prolonged latencies of SEPs suggest some damage to brain action [1]. MEP monitoring is able to directly monitor the function of the pyramidal tract, which can sensitively reflect the effects of cerebral ischemia and local operation traction on neural activity [2]. In general, it is suggested that no postoperative motor function deficit occurred when MEPs returned to normal after surgery. Thus, some authorities recommend that SEPs be monitored in combination with MEPs to ensure the functional integrity of somatosensory and motor conduction pathways. Obviously, monitoring SEPs alone is likely to preclude the detection of motor nervous system damage and monitoring MEPs alone tends to miss damage to sensory nerve pathways. SEPs and MEPs can complement each other to make nervous system monitoring complete and more robust.

A previous study revealed that intraoperative anesthetics have some significant effects on SEPs and MEPs. For instance, muscle relaxants have the greatest effect, followed by inhaled anesthetics and intravenous anesthetics [3, 4]. Total intravenous anesthesia is usually performed in neurosurgery when monitoring SEPs and MEPs; however, total intravenous anesthesia tends to cause considerable hemodynamic fluctuations in some clinical situations. Many anesthesiologists prefer to choose balanced anesthesia containing low-dose halogenated anesthetics (less than 1.0 minimum alveolar concentration (MAC)) in this approach [5]. No prospective controlled human study has compared the effects of desflurane and sevoflurane on SEPs and MEPs during extensive neurosurgery. Our goal was to test the hypothesis that the latencies and amplitudes of SEPs and MEPs would be inhibited at different concentrations by both desflurane and sevoflurane, and the desflurane would have a stronger inhibitory effect on SEPs and MEPs than sevoflurane at the same minimum alveolar concentration.

## Methods

### Ethics and patients

This study protocol was approved by the Institutional Ethics Committee of the Second Affiliated Hospital of Kunming Medical University (Kunming, China, approval number: PJ-2021-33). All methods were carried out in accordance with relevant guidelines and regulations and with CONSORT recommendations. The study was registered in the Chinese Clinical Trial Registry (Clinical Trials identifier: ChiCTR2100045504). Forty consecutive patients were enrolled in the study, and written informed consent was obtained from each patient. The inclusion criteria were adult patients (age range 18 to 60 years, ASA grade I or II, BMI range 20 to 25 kg/m2) scheduled to undergo general anesthesia for elective neurosurgery expected to last at least 3 h. All patients were neurologically normal at clinical examination, as determined preoperatively by the neurosurgeon. Exclusion criteria were patients less than 18 years old or more than 60 years old, an ASA grade greater than II, surgical time less than 3 h, preoperative neurological deficits or neuromuscular transmission dysfunction, cortical stimulation contraindications (seizures, pacemakers, previous skull surgery or implants), a preoperative left ventricular ejection fraction less than 30%, significant aortic regurgitation or cardiac arrhythmias, severe obstructive or restrictive ventilatory dysfunction, preoperative infection, emergency surgery, current pregnancy or lactation period, known allergy to halogenated anesthetics, and participation in another trial. Additionally, any patient who required unexpected emergency resuscitation during the operation, such as a patient who experienced massive bleeding (> 3000 ml), severe anaphylaxis, and cardiac arrest, was also excluded. Finally, any patient for whom monitoring (of measurements such as SEPs, MEPs and BIS) could not be successfully performed due to the nature of the surgical site was also excluded.

### Randomization, blinding, and data collection

The randomization of the study (1:1) was performed by the narcotic drug administrator in the Department of Anesthesia, using internet-based randomization software. The morning of the surgery, blinded halogenated anesthetics (visually identical plastic bottles of 100 ml) were delivered to the anesthesiologist in charge of the patient. Study anesthetics were only identified by the assigned patient number. Intraoperative electrophysiological data were analyzed and recorded by one neuroelectrophysiological specialist. Importantly, the neuroelectrophysiological specialist remained blinded to the anesthetic allocation and the inspiratory concentration when collecting data.

### Anesthetic protocol

All included patients were allowed solid foods up to 6 h before surgery and fluids up to 2 h before surgery. Patients received 1 to 2 mg midazolam (H10980025, Jiangsu Nhwa Pharmaceutical Company, China) intravenously after entering the operating room. Standard monitoring for this surgery included a one-lead electrocardiogram, pulse oximetry, noninvasive blood pressure, urine output, oropharyngeal temperature, invasive radial arterial pressure, central venous pressure, inspiratory and expiratory gas concentrations, and continuous electroencephalography (A-2000xp BIS monitor, Aspect Company, America) to monitor the depth of anesthesia. For the induction of anesthesia, intravenous 0.3 μg/kg sufentanil (090311, Hubei Yichang Renfu Pharmaceutical Company, China), 2 mg/kg propofol (GS298, Fresenius Kabi Company, Germany), 0.7 mg/kg rocuronium (1,050,213, Jiangsu Hengrui Pharmaceutical Company, China) were administered. After tracheal intubation, a bite block was used to protect the teeth and tongue. Mechanical ventilation with oxygen/air was performed, and Fio2 and respiratory parameters were adjusted to maintain SpO2 in 95-100% and PETC02 in 30-35 mmHg. Body temperature was kept between 36 and 37 degrees Celsius by thermal means. General anesthesia was maintained by intravenous pumping of propofol (75 to 125 μg/kg/min), and remifentanil (0.1 to 0.2 μg/kg/min) and volatile anesthetic either sevoflurane (0202, Marushi Pharmaceutical Company, Japan) or desflurane (4,474,432, Astra Zeneca Company, England) depending on anesthetic allocation. However, no further doses of neuromuscular blocking agents were subsequently used. Propofol and remifentanil concentrations were adjusted intraoperatively to target bispectral index (BIS) values between 40 and 60. All anesthetics were administered according to the patient’s actual weight. During the operation, blood pressure was maintained between 80 and 120% of the preoperative baseline level, and the heart rate was more than 50 times per min. Any incidence of low heart rate and low blood pressure was treated as appropriate with cardiovascular active drugs such as noradrenaline, ephedrine and atropine.

### Neurophysiological monitoring

All neurophysiological monitoring measurements were performed and recorded by the same trained neurophysiologist using the same equipment each time and without knowing which anesthetic method was chosen. Multipulse transcranial electric stimulation was achieved using the Cadwell Cascade TCS-1 evoked potential machine (Cadwell Laboratories, Kennewick, America). The machine delivered a constant voltage to evoke a compound muscle action potential, which was performed after craniotomy and incision of the meninges. The electrodes were placed in accordance with international EEG standards (international 10 to 20 system) [6].

SEPs were elicited by bilateral stimulation of the median nerves at the wrist in the upper limbs and the posterior tibial nerves at the ankle in the lower limbs using subdermal needle electrodes. Continuous single pulse electrical stimulation with a stimulation intensity of 10-35 mA was presented at a rate of 2-10 Hz. SEPs were recorded through needle electrodes placed over the cortex at the C3’ and C4’ areas, defining two channels: the Cz active electrode, in the midline of the scalp 2 cm behind the earlobe, and the midfrontal (Fpz) reference, approximately 10 cm before the sagittal line of two external auditory meatuses [7]. The electrode impedance was lower than 5.0 kΩ. The amplifier bandpass was 30 to 1000 Hz. An analysis time of 100 ms was used; for each SEP waveform, 100 to 300 sweeps were averaged. A first set of SEP recordings was obtained after the train-of-four twitch assessment (TOF) value was more than 90% and recordings were repeated more than 2 times to ensure data stability and repeatability. Stimulus intensity was adjusted above the motor threshold and was maintained at this level during surgery.

Neurogenic MEPs were obtained by stimulating the motor cortex through sterile platinum needle electrodes inserted by the neurosurgeons. Stimulating electrodes were placed 2 cm in front of the SEP cortical electrodes at the Cz area along the motor cortex [8]. The ground electrode was located at the sternum. A train of 5 square pulses, applied for 0.05-ms duration each, was delivered at an interstimulus interval of 2 ms (500 Hz). Stimulation of the upper limbs started at 250 V and that of the lower limbs started at 300 V; then, stimulation gradually increased until a reproducible MEP was elicited. Stimulation was then fixed at this threshold intensity for this study. The maximum output was limited to 450 V. MEP recordings were obtained with stainless steel hollow-bore subdermal 12-mm electrodes in the first dorsal interosseus and tibialis anterior bilaterally. As needle electrodes were used, the impedance of stimulation and recording was thereby low. Filters were set at 10 and 5000 Hz, and the analysis time was 100 ms. For each MEP waveform, 10 to 20 sweeps were averaged, and onset latency (the time from stimulus onset to the first negative deflection) and peak-to-peak amplitude of the initial MEP complex were determined [6].

After the anesthesia depth was stable, a train-of-four twitch assessment was performed using a stimulator at the ulnar nerve. The variation in myokymia induced by the abductor pollicis muscle was measured by a TOF-watch SX accelerometer with a pulse width of 200 s, frequency of 2 Hz, and current of 60 mA. Muscle relaxation recovered completely only when the TOF value (T4/T1) was greater than 90%. The first set of SEP and MEP recordings was obtained approximately 60 min after anesthesia induction. Then, the concentration of inhaled anesthetics was adjusted so that the end-expiratory concentration would be 0.3, 0.6 and 0.9 MAC successively. Higher concentrations of volatile anesthetic agents were not tested to avoid excessive decreases in blood pressure. Each concentration was maintained for at least 10 min, which was regarded as a steady state. After stopping the surgical operation, the latencies and amplitudes of all SEPs and MEPs at each concentration were recorded. SEP and MEP data were collected only during noncritical stages of the surgery and when no extrinsic disturbances, such as diathermy, were present. The measurements were analyzed by the neurophysiological technologist without knowledge of the inhalational agent or its respective MAC. In the end, we took the average of at least two values in each record.

### Statistical analysis

To obtain better statistics, we included 20 patients in each group, and the amplitudes and latencies of all SEPs and MEPs were recorded and analyzed. The normality of the data distribution was tested using the Kolmogorov-Smirnov test. SPSS statistics 21.0 software was used for statistical analysis. The data are presented as the mean and SD for amplitude and latencies of SEP and MEP signals. The paired sample T-test method was used for intragroup data comparison, while the independent sample T-test method was used for intergroup comparison. A probability value less than 0.05 was considered significant.

## Results

The 40 neurosurgical patients were monitored with intraoperative somatosensory evoked potentials and motor evoked potentials. There were no statistically significant differences in the preoperative baseline biological parameters, such as sex, age, weight, blood pressure, heart rate, PaO2, PETCO2, temperature and disease type (*P* > 0.05), at the introduction of each agent in either group. The mean infusion rate of propofol and remifentanil before the administration of each inhaled anesthetic being studied was kept standardized. Intraoperative combined monitoring of evoked potentials was successfully completed in all patients, without related complications (such as headache, seizures or nerve damage) caused by monitoring. Additionally, no neurological deficits occurred after the operation and all patients had normal postoperative neurological examinations results. The physiological data are listed in Table 1.

**Table 1:** Baseline biological parameters

Characteristics	Desflurane (*n* = 20)	Sevoflurane (*n* = 20)
Age (y)	41 ± 15	46 ± 11
Male/female (n)	12/8	10/10
Weight (kg)	65 ± 19	70 ± 21
Duration of surgery (min)	378 ± 94	408 ± 114
Mean arterial pressure (mm Hg)	80 ± 14	74 ± 10
Heart rate (b/min)	76 ± 17	72 ± 12
SpO2 (%)	99 ± 2	99 ± 1
PET CO2 (mm Hg)	33 ± 2	34 ± 3
Temperature (°C)	36.3 ± 0.5	36.5 ± 0.6
Propofol dose (mcg/kg/min)	91 ± 25	87 ± 21
Remifentanil dose (mcg/kg/min)	0.15 ± 0.2	0.14 ± 0.2
Type of disease (%)		
Acoustic neuroma	11 (55)	10 (50)
Intracranial aneurysm	3 (15)	4 (20)
The brain stem tumor	3 (15)	2 (10)
Frontal glioma	2 (10)	3 (15)
Parietal meningioma	1 (5)	1 (5)

No significant differences were found between groups (*P* > 0.05) for all parameters

The BIS values remained in the range of 40 to 60. In 40 patients who underwent an operation in this study, 8 patients had their intraoperative blood pressure increase and decrease by more than 20% the basic blood pressure range and then return to normal after treatment with deepening anesthesia, ephedrine or norepinephrine. Additionally, there were 5 patients whose heart rate was lower than 50 beats/min during the operation, and their heart rate returned to normal after treatment with atropine.

SEP and MEP signals were well preserved in patients who underwent neurosurgery under general anesthesia supplemented with desflurane or sevoflurane at concentrations of 0.3, 0.6 and 0.9 MAC. In each desflurane or sevoflurane group, the SEP amplitudes decreased, and the SEP latencies were prolonged significantly as the MAC increased (*P* < 0.05); only the SEPs of the upper limbs at 0.3 MAC in the sevoflurane group did not vary significantly (Table 2). In each desflurane or sevoflurane group, the amplitudes of MEPs decreased significantly as the MAC increased, and the latencies of MEPs were prolonged significantly at 0.9 MAC (*P* < 0.05) (Table 3).

**Table 2:** SEP latency and amplitude

Monitoring	Limbs	Groups	Baseline	0.3MAC	0.6MAC	0.9MAC
Latency (ms)	Upper limbs	Desflurane	17.35 ± 1.65	18.73 ± 1.72^1#^	20.01 ± 1.73^2#^	21.44 ± 2.03^3#^
		Sevoflurane	17.38 ± 1.53	17.51 ± 1.59	18.87 ± 1.63^2^	20.02 ± 1.74^3^
	Lower limbs	Desflurane	37.02 ± 2.25	39.24 ± 2.27^1#^	40.72 ± 2.01^2#^	42.35 ± 2.41^3#^
		Sevoflurane	36.97 ± 2.39	37.72 ± 2.21	39.19 ± 2.10^2^	40.82 ± 2.01^3^
Amplitude (μV)	Upper limbs	Desflurane	0.88 ± 0.35	0.79 ± 0.31^1#^	0.70 ± 0.33^2#^	0.53 ± 0.34^3#^
		Sevoflurane	0.88 ± 0.47	0.85 ± 0.30	0.78 ± 0.34^2^	0.63 ± 0.31^3^
	Lower limbs	Desflurane	0.82 ± 0.33	0.72 ± 0.41^1#^	0.59 ± 0.33^2#^	0.50 ± 0.31^3#^
		Sevoflurane	0.83 ± 0.31	0.79 ± 0.30	0.67 ± 0.28^2^	0.61 ± 0.25^3^

^1^*P* < 0.05 versus Baseline

^2^*P* < 0.05 versus 0.3MAC

^3^*P* < 0.05 versus 0.6MAC

^#^*P* < 0.05 versus Sevoflurane Group

**Table 3:** MEP latency and amplitude

Monitoring	Limbs	Groups	Baseline	0.3MAC	0.6MAC	0.9MAC
Latency (ms)	Upper limbs	Desflurane	28.12 ± 2.64	28.35 ± 2.69	29.50 ± 3.40	34.21 ± 6.64^3#^
		Sevoflurane	28.08 ± 2.87	28.41 ± 2.98	29.76 ± 4.03	32.13 ± 5.15^3^
	Lower limbs	Desflurane	43.63 ± 5.39	43.91 ± 5.99	45.03 ± 10.99	50.77 ± 10.06^3#^
		Sevoflurane	43.71 ± 5.64	43.87 ± 6.48	44.67 ± 6.33	47.53 ± 5.40^3^
Amplitude (μV)	Upper limbs	Desflurane	1920.3 ± 1762.1	1303.4 ± 1455.3^1^	590.4 ± 372.1^2#^	273.1 ± 239.5^3#^
		Sevoflurane	1847.5 ± 1655.0	1236.1 ± 1270.7^1^	891.0 ± 988.3^2^	407.2 ± 254.7^3^
	Lower limbs	Desflurane	1113.2 ± 1137.0	788.9 ± 734.3^1#^	444.7 ± 324.2^2#^	135.0 ± 221.8^3#^
		Sevoflurane	1123.6 ± 1127.5	931.3 ± 798.4^1^	719.9 ± 664.1^2^	321.6 ± 298.4^3^

^1^*P* < 0.05 versus Baseline

^2^*P* < 0.05 versus 0.3MAC

^3^*P* < 0.05 versus 0.6MAC

^#^*P* < 0.05 versus Sevoflurane Group

When monitoring SEPs, the latencies in both the upper and lower limbs in the desflurane group were significantly longer, and SEP amplitudes in the desflurane group were significantly lower than those in the sevoflurane group (*P* < 0.05) (Table 2). When monitoring MEPs, the amplitudes in the lower limbs in the desflurane group were significantly lower than those in the sevoflurane group at 0.3 MAC (*P* < 0.05); the amplitudes in both upper and lower limbs in the desflurane group were significantly lower than those in the sevoflurane group at 0.6 MAC (*P* < 0.05); and the latencies of both upper and lower limbs in the desflurane group were significantly longer and their amplitudes were significantly lower than those in the sevoflurane group at 0.9 MAC (*P* < 0.05) (Table 3).

Analyzing the measured results, we found that although we were still able to obtain reproducible SEPs and MEPs with multipulse stimulation in all 40 patients using either inhalational anesthetic agent, both desflurane and sevoflurane depressed SEPs and MEPs in a dose-dependent manner. In each desflurane or sevoflurane group, the amplitudes of SEPs and MEPs decreased, and the latencies of SEPs were prolonged significantly as the MAC increased. Only 0.3 MAC sevoflurane had no significant suppressive effect on either SEP latencies or amplitudes in the lower limbs. Under the same MAC conditions, it seemed that the inhibitory effect of desflurane was stronger than that of sevoflurane. The amplitudes of SEPs and MEPs in the desflurane group were significantly lower, and the latencies of SEPs were significantly longer than those in the sevoflurane group (*P* < 0.05). The inhibitory effect on MEP amplitudes in the upper limbs did not vary significantly, but only at 0.3 MAC, between the sevoflurane and desflurane groups (Tables 2 and 3).

## Discussion

Intraoperative neurophysiological monitoring has gradually become an indispensable and irreplaceable part of neurosurgery for improving surgical accuracy, minimizing iatrogenic injury, reducing surgical risk, and avoiding irreversible damage to the brain, spinal cord or other related structures. SEPs are potential changes recorded in the nerve trunk and central nervous system when an appropriate stimulation acts on any point of the peripheral sensory organs or sensory nerve pathways. To some extent, SEPs can reflect the functional state of specific body sensory afferent pathways, brain stem reticular structures and the brain cortex; these features are currently the most important means for monitoring the function of the brain spinal cord and nerve roots during neurosurgery. By intraoperative SEP monitoring, some risk factors, such as cerebral spinal cord injury and nerve root injury, can be detected in a timely manner to avoid permanent damage [1, 9]. MEPs are electric responses in the efferent paths, effectors or muscles that can monitor the function of the downward motor nerve conduction system after electrical or magnetic stimulation acts on motor central nerves, such as the brain functional area and spinal cord. MEPs are highly sensitive to injury to the brain and spinal cord and are highly sensitive to changes in motor function, which can immediately reflect the influences of ischemia, traction and local operation on central nervous function [10, 11].

There are many influencing factors in SEP and MEP monitoring during an operation, and we should pay attention to the occurrence of false positive or negative results. Midazolam can bind to the benzodiazepine receptor BZ sites in the cerebral cortex and then produce an inhibitory effect. However, midazolam has little effect on the monitoring of SEPs when the dose does not exceed 150 μg/kg [12, 13]. Muscle relaxants, which act on the neuromuscular junction, have a great impact on MEPs and make monitoring MEPs almost impossible. To avoid the effects of muscle relaxants, no more muscle relaxants were used after anesthesia induction during the experiment [13, 14]. The main action site of propofol is generally the GABA receptor/C-channel complex, which has a certain effect on the release of synaptic transmitters. The plasma concentration of propofol is recommended to be maintained between 120 and 200 μg/kg/min, which seldom exerts an effect on the monitoring of SEPs and MEPs [13, 15, 16]. In our study, the blood concentration of propofol was adjusted within this range. In addition, opioids scarcely have effects on SEPs and MEPs [3, 16]; and furthermore, we adjusted the remifentanil blood concentration to maintain the BIS value between 40 and 60. By increasing the activity of inhibitory amino acids (GABAs) and reducing the activity of excitatory amino acids (NMDAs), inhaled anesthetics can inhibit neuron conduction and synaptic transmission and effect the production of SEPs and MEPs [17]. Furthermore, the depth of anesthesia has an uncertain influence, positive or negative, on SEPs and MEPs [18, 19]. In our study, it barely caused confusion about the results maintaining a BIS value between 40 and 60 for the surgical requirements [19]. Other confounding factors, such as temperature, blood pressure, surgical instruments and surgical operations, also have some uncertain influences on SEPs and MEPs. In this study, to minimize interference when monitoring SEPs and MEPs, invasive blood pressure was continuously monitored to maintain blood pressure between 80 and 120% of the basic blood pressure and a heart rate of more than 50 beats/min; cardiovascular active drugs were used when necessary. At the same time, body temperature was maintained between 36 and 37 °C.

SEP and MEP waveforms could be detected in all 40 cases of surgery at 0.3, 0.6 and 0.9 MAC, indicating that the monitoring of SEPs and MEPs could be carried out successfully when the inhalation concentration of desflurane and sevoflurane did not exceed 1.0 MAC. However, once the inhalation concentration of anesthetics reached 0.9 MAC, the amplitudes of SEPs and MEPs significantly decreased, and the latencies were significantly prolonged, suggesting that inhaled anesthetics have a significantly stronger inhibition effect on SEPs and MEPs when exceeding 0.9 MAC. The abnormal standard of SEPs is a latency extension greater than 10% or an amplitude decrease greater than 50% compared with the baseline. The SEP latency extension of desflurane on the upper limbs at 0.6 and 0.9 MAC exceeded 10% of the base value, and the SEP latency extension of sevoflurane at 0.9 MAC on both the upper and lower limbs exceeded 10% of the base value, indicating that the inhalation concentration of desflurane should not exceed 0.6 MAC and the inhalation concentration of sevoflurane should not exceed 0.9 MAC during SEP monitoring. The traditional MEP measurement that signifies a warning is a 50% decrease in amplitude or a 2.5 ms extension of latency. The MEP amplitude decrease in both upper and lower limbs in response to desflurane at 0.6 and 0.9 MAC was more than 50% of the base value, and MEP amplitudes in response to sevoflurane at 0.6 and 0.9 MAC in the upper limbs decreased by more than 50% of the base value, indicating that the inhalation concentration of desflurane or sevoflurane should not exceed 0.6 MAC during the monitoring of MEPs. Therefore, when monitoring SEPs combined with MEPs, the concentration of inhaled anesthetics is preferably maintained below 0.6 MAC to obtain more accurate monitoring results and not disturb the judgment of the operator.

The results of this research demonstrated that the changing trends of SEPs and MEPs were consistent with the inhibitory effects of desflurane and sevoflurane in a dose-dependent manner. Studies have shown that inhaled anesthetics not only increase the activity of GABA receptor, but also decrease the activity of NMDA receptor, whose inhibitory effect changes step by step with changes in the end-expiratory anesthetic concentration [20, 21]. However, at 0.3 MAC, sevoflurane had no significant suppressive effect on either SEP latencies or amplitudes in the lower limbs compared with baseline. This outcome may suggest that the use of 0.3 MAC sevoflurane (but not desflurane) provided good SEP recordings acceptable for clinical interpretation, due to the inapparent influences of inhaled anesthetics when at a low concentration. Similar results were demonstrated by Baker A et al. [20] who showed that the SEP latencies and amplitudes changed little when the concentration of anesthesia was less than 0.4 MAC.

By comparing the desflurane group and sevoflurane group, we found that the inhibitory effect of desflurane was significantly stronger than that of sevoflurane at the same concentration, which may be considered a reason that desflurane inhibits GABA metabolism more strongly than sevoflurane at the synaptic level. Sevoflurane could be given priority when selecting inhaled anesthetic agents during intraoperative SEP and MEP monitoring. However, the inhibitory effect on MEP amplitudes of the upper limbs at 0.3 MAC between the sevoflurane and desflurane groups did not vary significantly, considering that they had similar low inhibitory effects on GABA metabolism at the synaptic level. When at a low concentration (such as 0.3 or 0.6 MAC), desflurane and sevoflurane seem to have no significant suppressive effect on MEP latencies in either the upper or lower limbs. Similar results were demonstrated by Chin Ted Chong et al. [22], who showed that MEP latency changes little when the concentration of anesthesia was less than 0.7 MAC. The lower limbs appear to be more sensitive to anesthetic-induced depression compared with the upper limbs, as the magnitude of amplitude depression and latency extension of SEPs and MEPs is overall greater for lower limbs than for upper limbs. This finding is consistent with a study by Chin Ted Chong et al. [22], which suggested that the reason for this outcome is related to the difference in cortical spinal cord drive mechanisms between upper and lower limbs [23].

However, there are some limitations in our research due to the constraints of experimental conditions. For example, Ushirozako H et al. [24] demonstrated in a recent study that long-term exposure to anesthetics requires a higher stimulus threshold to elicit MEP responses, which is different from a dose-dependent inhibitory effect. This occurrence is a phenomenon called “anesthetic fade” and is indeed a concern in long case studies. This phenomenon is a flaw in our study because we usually chose large major neurosurgical operations for neurophysiological monitoring, which tended to provide sufficient time to complete these research measurements without unnecessarily prolonging the surgical procedure or interfering with the essential monitoring. In addition, muscle relaxants are known to have a significant effect on MEP monitoring and even make it impossible to perform. Although muscle relaxants were not used in this experiment except for anesthesia induction, sevoflurane and desflurane may have partial neuromuscular block effects under 0.9 MAC [25]; these effects are likely to influence MEP monitoring during operation. All patients studied had normal neurological examination; hence, these results may not be applicable to those with preexisting deficits.

## Conclusions

This study is the first to comprehensively compare the effects of two inhaled anesthetics, sevoflurane and desflurane, on somatosensory evoked potentials and motor evoked potentials. The results demonstrated that SEP and MEP monitoring could be carried out successfully under the usual doses of sevoflurane and desflurane for general anesthesia. However, to obtain more accurate monitoring results and to avoid interfering with the judgment of the operator, the concentration of inhaled anesthetics was recommended to be below 0.6 MAC. Furthermore, the inhibitory effect of desflurane was significantly stronger than that of sevoflurane at the same concentration. When monitoring SEPs or MEPs during operation, it is recommended that sevoflurane be given priority. Finally, monitoring both the upper and lower limbs simultaneously is recommended to avoid confounding factors.

## Data Availability

The datasets used and analyzed during the current study are available from the corresponding author upon reasonable request.

## Abbreviations

SEP: Somatosensory-evoked potential
MEP: Motor-evoked potential
MAC: Minimum alveolar concentration
ECG: Electrocardiography
SpO2: Pulse oxygen saturation
ETCO2: End-tidal CO2
TOF: Train-of-four twitch assessment
GABA: Gamma-aminobutyric acid
NMDA: N methyl D aspartate
